# Supplementary material for: A minority of final stacks yields superior amplitude in single-particle cryo-EM
Source: Nat Commun. 2023 Dec 10;14:7822. doi: 10.1038/s41467-023-43555-x (PMC10711021; doi:10.1038/s41467-023-43555-x)
Supplement: Supplementary file 1 — Supplementary Information [file 41467_2023_43555_MOESM1_ESM.pdf]

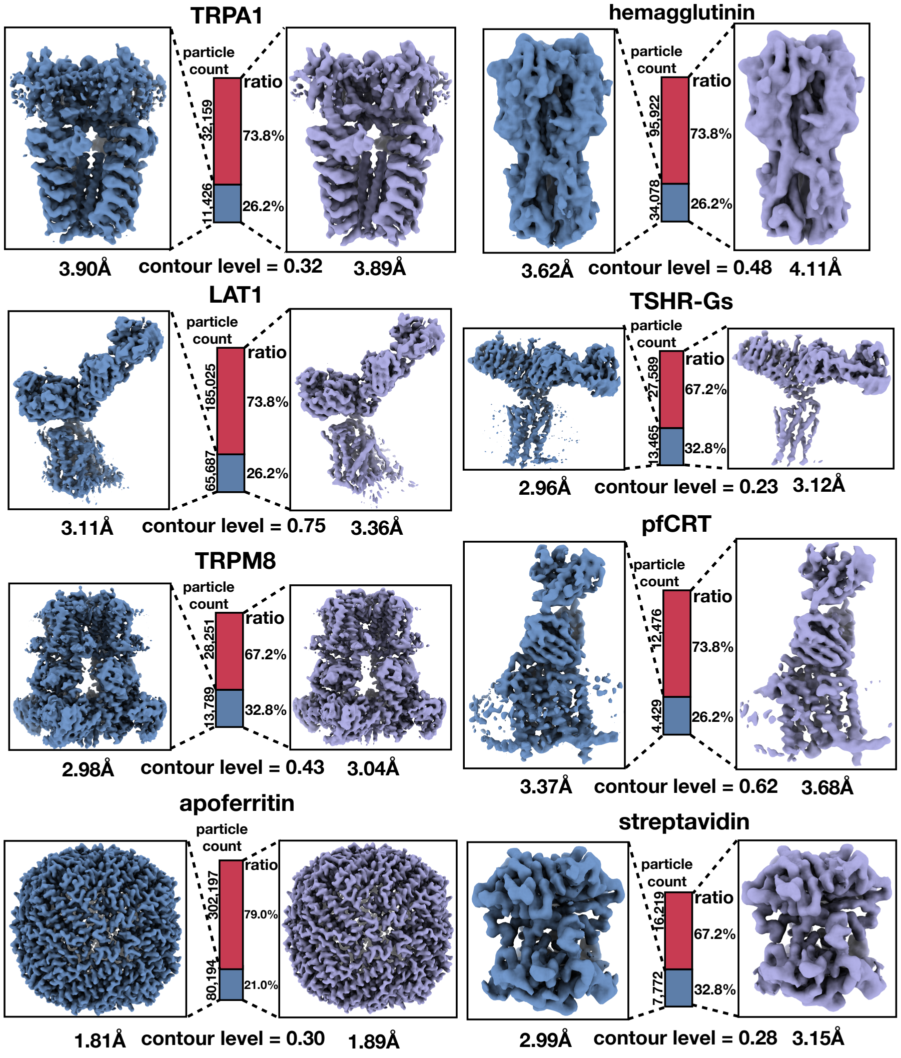

Supplementary Figure 1: **Raw density maps derived from both the CryoSieve-retained particles and the complete set of particles in the final stacks.** For all eight experimental datasets, we juxtaposed the raw density maps—without B-factor sharpening—from both the CryoSieve-retained particles (depicted in steel blue) and the full set of particles in the final stack (shown in medium purple). These were derived from CryoSPARC's *ab initio* reconstruction, with the exception of streptavidin where local refinement was utilized. The equivalent contour level was applied for each protein respectively, as indicated at the base of each ratio bar.

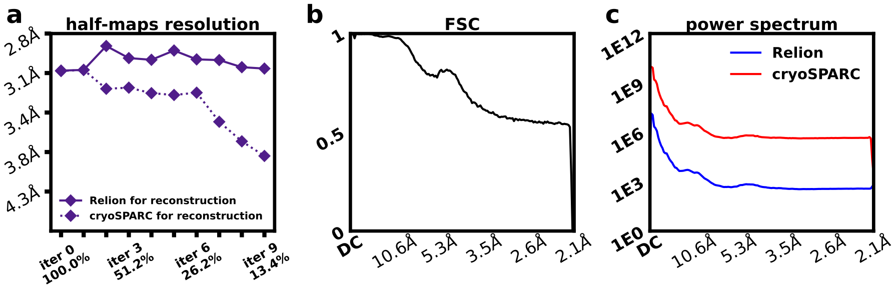

Supplementary Figure 2: **Performance comparison of RELION and CryoSPARC used as the reconstruction module in CryoSieve.** **a**, The half-maps FSC resolutions are depicted. We utilized the TRPM8 datasets for our testing. The default retention ratio is set at 0.8, with a starting frequency of 40Å and an ending frequency of 3Å. **b**, The FSC curve between the density maps reconstructed using RELION and CryoSPARC. **c**, Power spectra of the density maps reconstructed using RELION and CryoSPARC are overlaid on the same plot for direct comparison. Identical particles and poses (from TRPM8 dataset) were employed for both reconstructions in **b** and **c**.

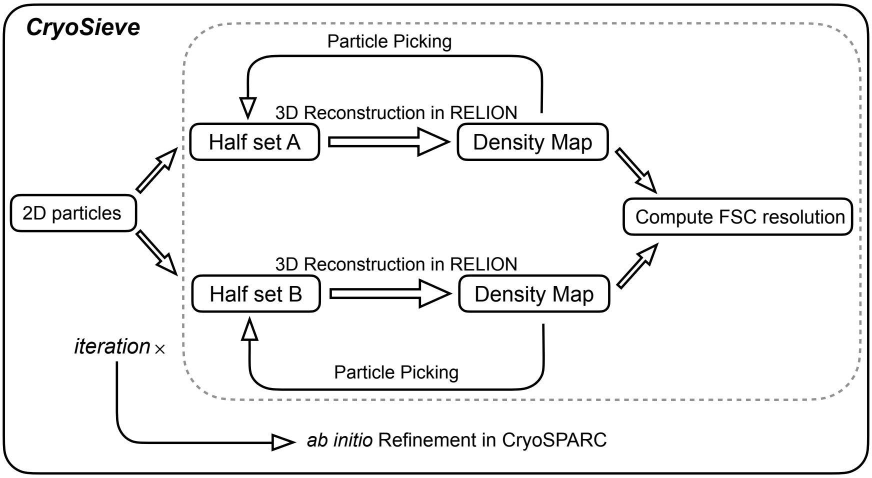

Supplementary Figure 3: **Flow chart scheme for CryoSieve.** CryoSieve operates through multiple iterations. Each iteration comprises both density map reconstruction and particle sieving.

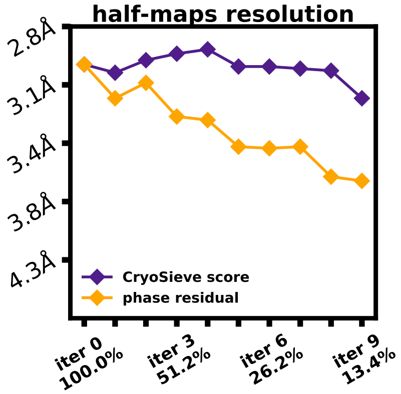

Supplementary Figure 4: **Performance comparison of CryoSive score and phase residual used as the particle selection module in CryoSieve.** The half-maps FSC resolutions are depicted. We utilized the TRPM8 datasets for our testing. The default retention ratio is set at 0.8, with a starting frequency of 40Å and an ending frequency of 3Å.

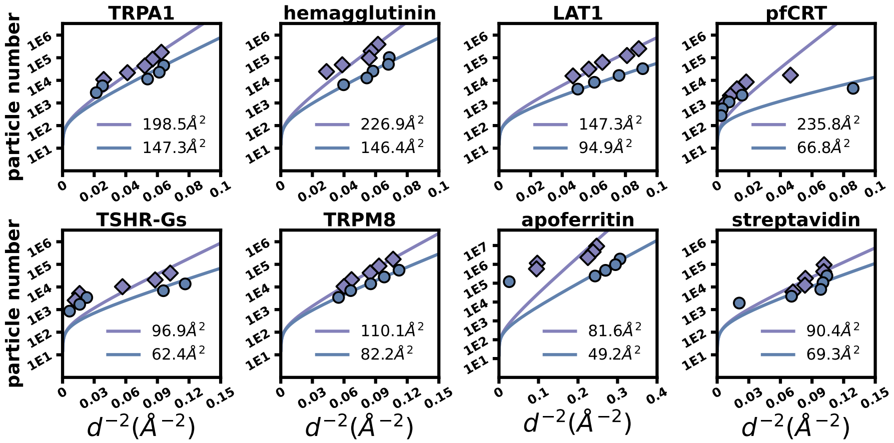

Supplementary Figure 5: Scatter points representing the reciprocal of the square of the resolution against the log scale of particle number are depicted: steel blue circles for particles retained by CryoSieve and medium purple diamonds for all particles in the final stack. Moving from top to bottom, each point represents half the particles of the previous one. The resolutions were determined by CryoSPARC's refinement. B-factors were determined using a least-squares approximation of the measured points, as shown by the fitting curves. The estimated Rosenthal and Henderson's B-factors are indicated in the legends: steel blue represents particles retained by CryoSieve, while medium purple denotes all particles in the final stack.

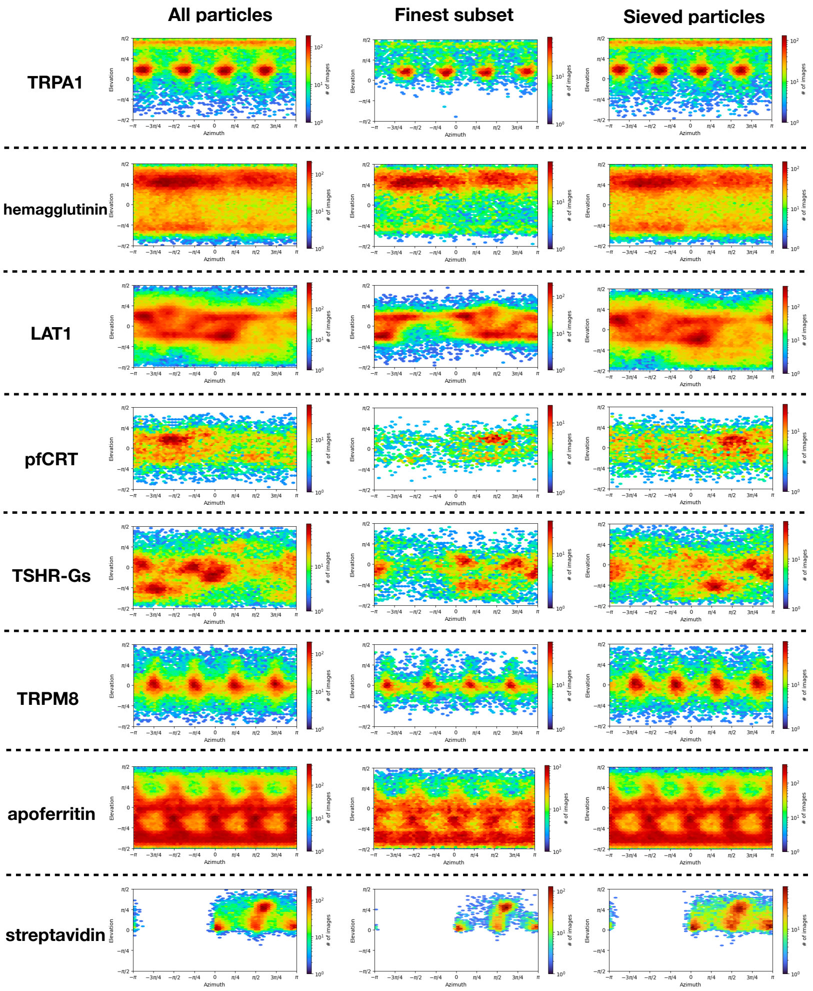

Supplementary Figure 6: **Comparision of angle distribution before and after CryoSieve.** Pose distribution reported by CryoSPARC are plotted.

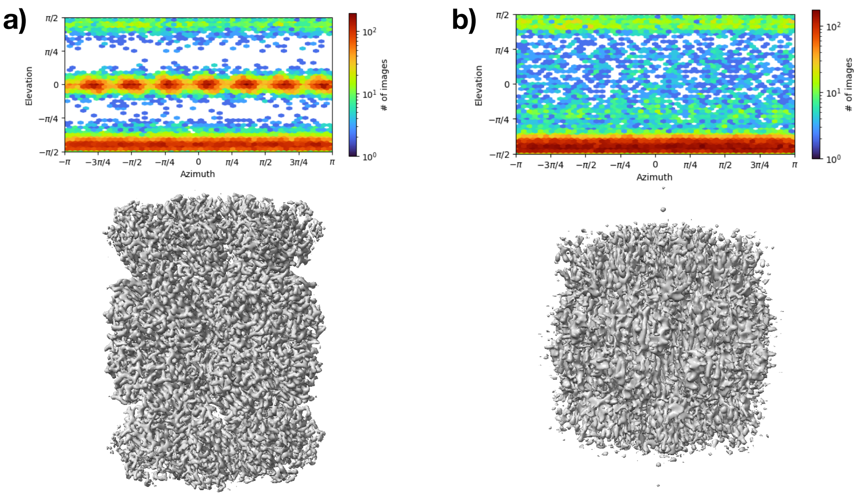

Supplementary Figure 7: **Comparison of pose distributions and density maps derived from retained particles using CryoSieve and cisTEM reveals variations in the proteasome dataset subject to differing levels of radiation damage. a**, CryoSPARC's pose distribution (upper) and density maps (lower) are depicted. For this analysis, the sixth iteration of CryoSieve was selected, where 26.2% of particles were retained. **b**, The pose distribution (upper) and density maps (lower) are shown, retaining the same number of particles using the cisTEM score.

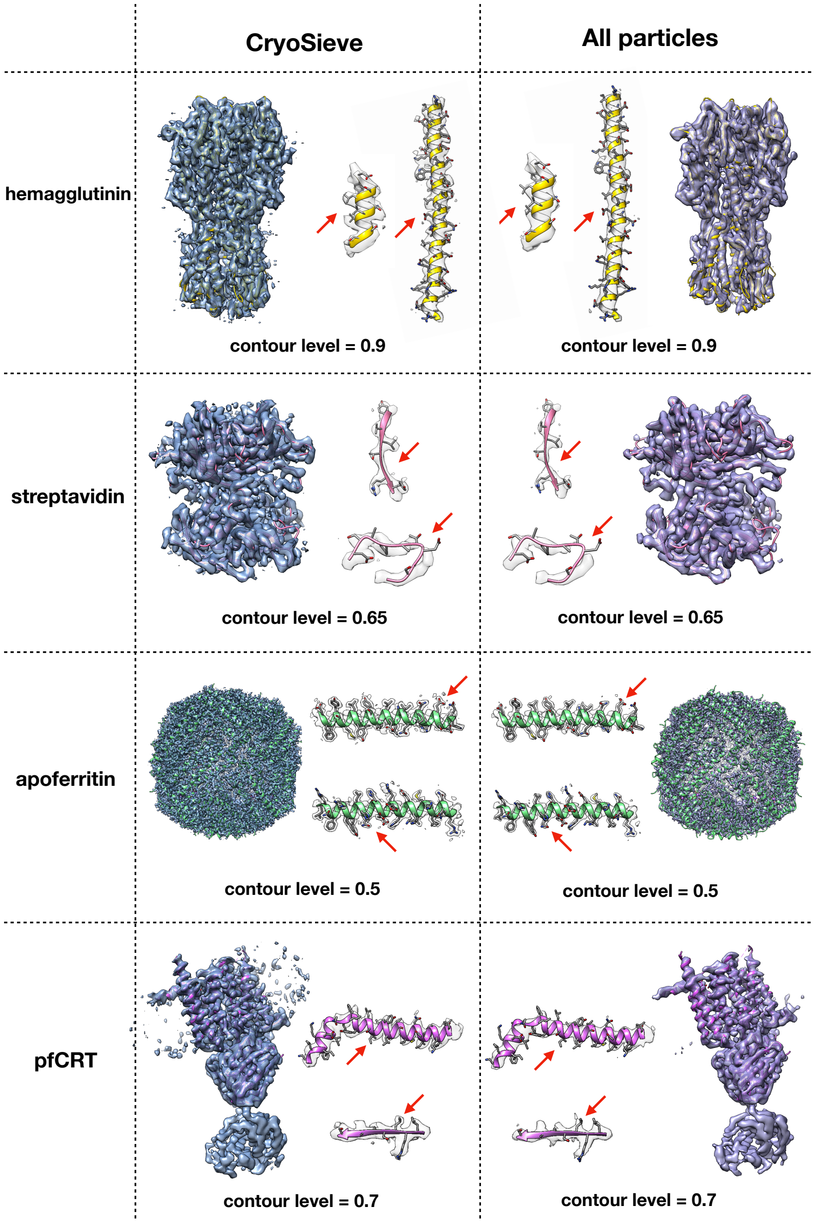

Supplementary Figure 8: **Detailed comparisons of local cryo-EM densities before and after cryoSieve.** Local densities before and after applying CryoSieve were compared. The B-factors used for sharpening were identical to those in Figure 1 of the main text. Red arrows highlight the improved regions.

Supplementary Material I: Theoretical analysis of CryoSieve score.

Assuming the noise in particles follows from the Gaussian distribution, we proved the probability of assigning a higher CryoSieve score to correct particles was close to 1, as opposed to typical incorrect particles in cryo-EM.

We consider two populations of image patches: good patches, which agree with our 3D reference, and bad patches, which have random phased high-frequency components or have incorrect estimated imaging parameters such as rotation angle, in-plane translation, and CTF. Let b_1, b_2 denote random samples from good and bad particle patches, respectively. The effectiveness of CryoSieve score could be written in the following formulation: there is a high probability that CryoSieve score of b_1 is higher than b_2, i.e.,

$$\mathbf{Pr}[g_1 > g_2] > 1 - \eta \quad \text{for a small constant } \eta, \tag{1}$$

where

$$g_i = \|Hb_i\|_2^2 - \|H(b_i - \tilde{A}_i x)\|_2^2 = \|b_i\|_H^2 - \|b_i - \tilde{A}_i x\|_H^2. \tag{2}$$

Here, H is the chosen high-pass operator, $\tilde{A}_i$ is the estimated imaging operator of b_i, x is the reconstructed density map. The next theorem characterizes the probability in (1).

Theorem 1 *Let $b_i = A_i x_i + \varepsilon_i, i = 1, 2, \cdots$, where A_i is the true (oracle) imaging operator of particle b_i, x_i is the oracle 3D density map for particle b_i, and ε_i is the noise and satisfies $\varepsilon_i \overset{\text{i.i.d}}{\sim} N(0, \sigma^2 I)$. Let g_i be the CryoSieve score of the corresponding particle b_i as given by (2), we have*

$$\mathbf{Pr}_{\varepsilon_1,\varepsilon_2}[g_1 > g_2] =$$

$$1 - \mathrm{erf}\left(- \frac{\|A_1 x_1 - \tilde{A}_1 x\|_H^2 - \|A_1 x_1\|_H^2 - \|A_2 x_2 - \tilde{A}_2 x\|_H^2 + \|A_2 x_2\|_H^2}{2\sigma\sqrt{\|\tilde{A}_1 x\|_H^2 + \|\tilde{A}_2 x\|_H^2}} \right), \tag{3}$$

where $\mathrm{erf}(\cdot)$ *is the Gauss error function.*

Proof For each particle b_i, it is observed that

$$\begin{aligned} g_i &= \|b_i\|_H^2 - \|b_i - \tilde{A}_i x\|_H^2 = 2\langle b_i, \tilde{A}_i x\rangle_H - \|\tilde{A}_i x\|_H^2 \\ &= 2\langle A_i x_i + \varepsilon_i, \tilde{A}_i x\rangle_H - \|\tilde{A}_i x\|_H^2 = 2\langle A_i x_i, \tilde{A}_i x\rangle_H + 2\langle \varepsilon_i, \tilde{A}_i x\rangle_H - \|\tilde{A}_i x\|_H^2 \\ &= \|A_i x_i\|_H^2 + 2\langle \tilde{A}_i x, \varepsilon_i\rangle_H - \|A_i x_i - \tilde{A}_i x\|_H^2. \end{aligned}$$

By the linearity of ε_i, we know

$$\begin{aligned} g_1 - g_2 \sim N\big(&- \|A_1 x_1 - \tilde{A}_1 x\|_H^2 + \|A_1 x_1\|_H^2 + \|A_2 x_2 - \tilde{A}_2 x\|_H^2 - \|A_2 x_2\|_H^2, \\ &4(\|\tilde{A}_1 x\|_H^2 + \|\tilde{A}_2 x\|_H^2)\sigma^2\big), \end{aligned}$$

which implies (3). □

There may be many kinds of factors contributing to an incorrect particle. In this study, we focus on two specific types of errors: those resulting from high-frequency components random phased, and those caused by inaccurate estimation of imaging parameters such as rotation angle, in-plane translation, and CTF parameters. The first type of erroneous particles involved randomly damaging high-frequency components in the Fourier domain by introducing random phases, while the second type of erroneous particles is associated with errors in parameter estimation.

For random phase error, we assume that the reconstructed density map and the estimated pose parameters are precise, but the erroneous particle is imaged from a radiation damaged molecule. In particular, we assume in Theorem 1 that $A_i = \tilde{A}_i$, $x_1 = x$ while $x_2 = Rx$ where R is a radiation damaging operator. In cryo-EM, we can model radiation damage by a high-frequency band random phasing operator. Then, we have

$$\mathbf{Pr}[g_1 > g_2] = 1 - \text{erf}\left(\frac{-\|A_1x\|_H^2 - \|A_2x - A_2Rx\|_H^2 + \|A_2Rx\|_H^2}{2\sigma\sqrt{\|A_1x\|_H^2 + \|A_2x\|_H^2}}\right). \quad (4)$$

Moreover, assuming that $A_1 = A_2 = A = T \circ C \circ P$, where P is the projection operator, C is the CTF convolution operator, and T is the in-plane translation operator, we know (4) implies

$$\begin{aligned}\mathbf{Pr}[g_1 > g_2] &= 1 - \text{erf}\left(\frac{-\|Ax\|_H^2 - \|A(I-R)x\|_H^2 + \|ARx\|_H^2}{2\sqrt{2}\sigma\|Ax\|_H}\right)\\ &= 1 - \text{erf}\left(\frac{-\|TCPx\|_H^2 - \|TCP(I-R)x\|_H^2 + \|TCPRx\|_H^2}{2\sqrt{2}\sigma\|TCPx\|_H}\right)\\ &= 1 - \text{erf}\left(\frac{-\|CPx\|_H^2 - \|CP(I-R)x\|_H^2 + \|CPRx\|_H^2}{2\sqrt{2}\sigma\|CPx\|_H}\right)\\ &= 1 - \text{erf}\left(\frac{-\|\hat{H}\hat{C}\hat{S}\hat{x}\|^2 - \|\hat{H}\hat{C}\hat{S}(I-\hat{R})\hat{x}\|^2 + \|\hat{H}\hat{C}\hat{S}\hat{R}\hat{x}\|^2}{2\sqrt{2}\sigma\|\hat{H}\hat{C}\hat{S}x\|}\right).\end{aligned}$$

Here, $\hat{S}$ is the Fourier transform of P, which denotes the slice operator in the Fourier domain according to the central slice theorem. Assume the frequency of the random phase is equal to the high-pass frequency, it has

$$\begin{aligned}\mathbf{Pr}[g_1 > g_2] &= 1 - \text{erf}\left(-\frac{\|I-\hat{R}\|_{Frob}^2\|\hat{H}\hat{C}\hat{S}\hat{x}\|}{2\sqrt{2}\sigma}\right)\\ &= 1 - \text{erf}\left(-\frac{\|I-\hat{R}\|_{Frob}^2\sqrt{f}\|\hat{C}\hat{S}\hat{x}\|}{2\sqrt{2}\sigma}\right)\\ &= 1 - \text{erf}\left(-\frac{\|I-\hat{R}\|_{Frob}^2 N\sqrt{f\cdot\text{SNR}}}{2\sqrt{2}}\right),\end{aligned}$$

where $f = \frac{\|\hat{H}\hat{C}\hat{S}\hat{x}\|^2}{\|\hat{C}\hat{S}\hat{x}\|^2}$ is the fraction of high-frequency band energy, $\mathrm{SNR} = \frac{\|\hat{C}\hat{S}\hat{x}\|^2}{N^2\sigma^2}$ is the signal-to-noise ratio, and N is the box size of the particle.

Another type of incorrect particle is those with an inaccurate estimation of imaging parameters, such as incorrect rotation angle, in-plane translation, and CTF parameters. In particular, we assume that $x_1 = x_2 = x$, $A_1 = \tilde{A}_1$ while $A_2 \neq \tilde{A}_2$ in Theorem 1. In this case, (2) is reduced to

$$\mathbf{Pr}[g_1 > g_2] = 1 - \operatorname{erf}\left(\frac{-\|A_1 x\|_H^2 - \|A_2 x - \tilde{A}_2 x\|_H^2 + \|A_2 x\|_H^2}{2\sigma\sqrt{\|A_1 x\|_H^2 + \|\tilde{A}_2 x\|_H^2}}\right).$$

Similarly, we assume that $A_1 = A = T \circ C \circ P$ and $\tilde{A}_2 = \tilde{A}$ is the incorrectly estimated image operator which involves three cases: $\tilde{A} = \tilde{T} \circ C \circ P$ (transition error), $\tilde{A} = T \circ \tilde{C} \circ P$ (CTF error), $\tilde{A} = T \circ C \circ \tilde{P}$ (projection angle error). Then, we have

$$\mathbf{Pr}[g_1 > g_2] = 1 - \operatorname{erf}\left(-\frac{\|H(A - \tilde{A})x\|_2^2}{2\sigma\sqrt{\|HAx\|_2^2 + \|H\tilde{A}x\|_2^2}}\right). \quad (5)$$

We conducted several numerical experiments to test the validity of the CryoSieve score with (4) and (5). Assume that x is the reconstructed density map from all particles of eukaryotic cyclic nucleotide-gated channel (CNG), with the resolution of 3.5Å. Let correct particles be the projection of the reconstructed map followed by the CTF convolution, while the incorrect particles were obtained by disturbing the correct particles in the following ways: random phase high-frequency component, incorrect rotation angle, incorrect translation, and incorrect CTF estimation, respectively. The cutoff frequency of the highpass operator H was set to 15Å. For two typical SNRs, we plotted the probability versus the incorrectness of particles, denoted as the disturb of defocus in CTF, the frequency of high-frequency random phase, the in-plane translation error and the disturb of projection angle, respectively (Supplementary Figure 9). The figure shows that the CryoSieve score achieves high accuracy for typical incorrect particles in cryo-EM, even at an extremely low SNR of 0.001.

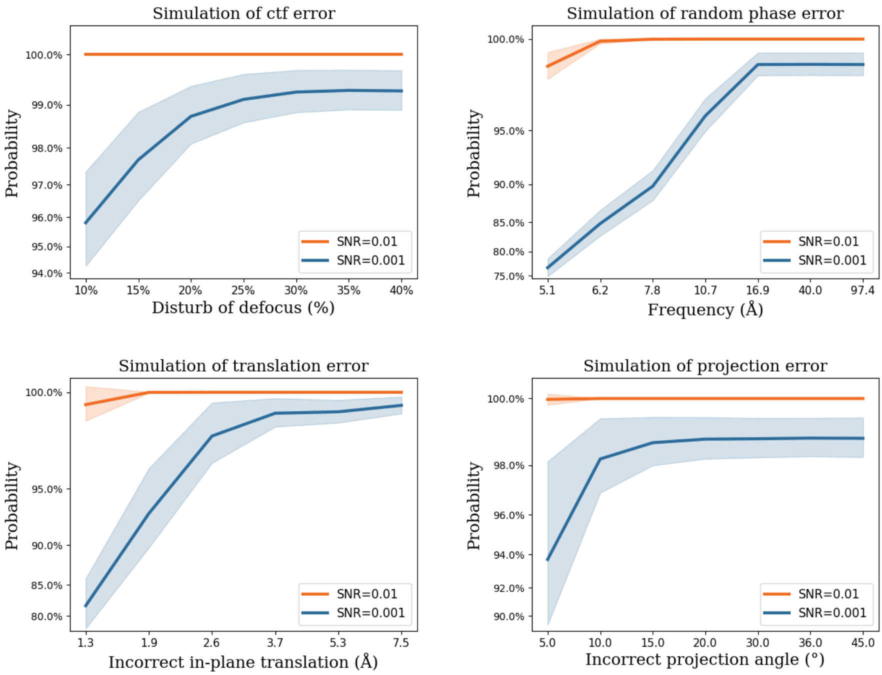

Supplementary Figure 9: **Theoretical probability of correct classification of simulated incorrect particles.** Incorrect particles with random phased high-frequency component, incorrect rotation angle, incorrect in-plane translation and incorrect CTF estimation were generated, respectively. The probability of assigning higher CryoSieve scores to correct particles could be writen as a function of the incorrectness of bad particles. The x-axis represents the degree of incorrectness for simulated bad particles, increasing from left to right. The orange and blue curves depict the mean probability of 10000 independent simulation experiments for different SNRs. Error bars represent the standard deviation of the same simulation experiments, with rotation angle of the correct particles randomly sampled from $SO(3)$ (light orange area, light blue area).

Supplementary Material II: Comparison between CryoSieve and cisTEM

To analyze the properties of the cisTEM score in the eight experimental datasets, we split the particles into two subsets, preserving the splitting in the original final stacks. We independently ran *Auto Refine* of cisTEM v1.0.0-beta with default parameters for each half set, resulting in a cisTEM score for each particle image. The particles in each half set were sorted in descending order of the cisTEM score, and particles with higher scores were selected, with the number of selected particles equivalent to that of CryoSieve. Finally, the selected particles were used for 3D density map generation by *ab initio* reconstruction of CryoSPARC, with splits preserved from input alignments by disabling "`Force re-do GS split`". We followed the same procedure and analysis setting as described in the Methods section.

Supplementary Material III: Comparison of performance among CryoSieve, non-alignment classification, NCC, and AGC based on varying simulated errors in particle datasets.

Datasets with pose and CTF estimation errors were constructed using simulated incorrect poses and CTF parameters. The TRPM8 dataset served as the basis for this generation.

For simulating incorrect orientation estimation, the orientation of 50% of the particles was perturbed, with rotations sampled from a uniform distribution in the rigid-body rotation space. To simulate inaccurate translation estimation, translations of particles were adjusted based on samples from a uniform distribution ranging between 1 and 3 pixels. Lastly, to simulate incorrect CTF parameter estimation, the CTF parameters for 50% of the particles were modified to introduce approximately a 30% variation (uniformly distributed between 15% and 45%) in defocus.

Supplementary Table 1: Sieving-out accuracy of various sorting algorithms based on varying simulated errors in particle datasets.

dataset	CryoSieve	non-alignment classification	NCC	AGC
incorrect orientation	97.16%	99.83%	96.67%	55.82%
incorrect translation	91.49%	92.89%	66.94%	50.04%
incorrect CTF parameter	91.77%	91.95%	65.63%	50.20%

CryoSieve, non-alignment classification, NCC, and AGC were tested using these simulated datasets. For both CryoSieve and NCC, the third iteration was selected, as during this iteration, nearly half (51.2%) of the particles are retained. For non-alignment classification and AGC, the default settings of these two algorithms were utilized. The accuracy of these methods in sieving out various simulated errors can be found in Supplementary Table 1.

Supplementary Material IV: Performance AGC on eight experimental datasets.

AGC, which is designed to remove Incorrectly oriented particle, was carried out using Scipion v3.0.12, following the instructions provided in its user manual. The initial volumes required for Scipion's AGC algorithms were the reconstructed maps of all particles in final stack using RELION v4.0-beta-2, applied with a mask identical to that used in CryoSieve and NCC. The "`symmetry group`" parameter of AGC was set to the corresponding symmetry of the structure, while the remaining parameters were set to their default values. Since the number of particles in the LAT1 dataset is too large to perform AGC without reporting error, we partitioned them into quarter-datasets and performed AGC algorithm within each subset. The remaining particles in the quarter-datasets were used for *ab initio* reconstruction in CryoSPARC. The number of particles in the apoferritin dataset is also too large to perform AGC without reporting error, so we performed AGC algorithm within a subset containing 20,000 particles for apoferritin. The remaining particles in the subset were used for *ab initio* reconstruction in CryoSPARC. We reported the metrics before and after AGC of this subset for apoferritin in Supplementary Table 1. Particle removal of other datasets were performed using all particles in final stacks as a whole.

Supplementary Table 2: Performance of AGC on eight experimental datasets.

dataset	AGC retaining ratio	model to map FSC resolution	half maps FSC resolution	Q-score of raw map	Q-score of sharpened map
		AGC / the original final stack			
TRPA1	93.74%	4.34Å/4.34Å	4.05Å/3.89Å	0.39/0.41	0.45/0.46
hemagglutinin	94.58%	4.53Å/4.47Å	4.06Å/4.11Å	0.41/0.41	0.34/0.35
LAT1	82.46%	3.88Å/3.88Å	3.38Å/3.36Å	0.51/0.51	0.50/0.50
pfCRT	89.48%	4.03Å/3.88Å	3.87Å/3.68Å	0.45/0.50	0.47/0.51
TSHR-Gs	77.46%	3.70Å/3.65Å	3.23Å/3.12Å	0.46/0.47	0.46/0.46
TRPM8	91.54%	3.49Å/3.49Å	3.15Å/3.04Å	0.56/0.60	0.52/0.53
apoferritin	65.03%	2.26Å/2.20Å	2.12Å/2.03Å	0.83/0.84	0.85/0.86
streptavidin	87.80%	10.37Å/3.46Å	4.80Å/3.15Å	0.13/0.58	0.12/0.61

Supplementary Material V: Performance of nonalignment classification on eight experimental datasets.

To compare the performance of CryoSieve with that of nonalignment classification, we applied nonalignment classification to sieve particles in the final stack. The particles were divided into four classes, and only particles belonging to the class with the highest resolution were retained. Subsequently, we used CryoSPARC to perform ab initio refinement on this selected group of particles.

For five out of eight datasets, the retention rate using non-alignment classification was above 90%, implying that the maps produced from the preserved particles either maintained their original quality or suffered a degradation (Supplementary Table 3). When non-alignment classification was employed on hemagglutinin, LAT1, and apoferritin, it culled less than half of the particles, leading to a modest resolution improvement (Supplementary Table 3). We compared the quality of density maps of these three datasets derived from non-alignment classification with those reconstructed from CryoSieve's finest subsets (Supplementary Table 4). Based on our findings, the enhancement from non-alignment classification is markedly inferior to the results obtained with CryoSieve.

In addition, we conducted variations in several parameters across all eight experimental datasets: the number of classes (K), the regularization parameter (tau2_fudge), and the number of iterations used in nonalignment classification. In this process, particles were categorized into various classes, with only those in the highest-resolution class being retained for further analysis. Specifically, we tested the configurations where the number of classes (K) were set to 20 and 40, the number of iterations (iter_num) were 40 and 80, and tau2_fudge values were chosen as 6 and 8. We selected the particle with the highest FSC resolution of the reconstruction density map from these eight possible combinations of paraemters and reported them. The box size of TSHR-Gs was 448, the particle number of LAT1 and apoferritin were 250,712 and 382,391, our machine run out of memory for some parameters of these datasets, so the iteration number of TSHR-Gs, LAT1, and apoferritin were set to 25. Subsequently, we used CryoSPARC to perform ab initio refinement on this selected group of particles. The results are shown in Supplementary Table 5 and Supplementary Table 6.

Supplementary Table 3: Performance of nonalignment classification on eight experimental datasets.

dataset	nonalignment classification retaining ratio	model to map FSC resolution	half maps FSC resolution	Q-score of raw map	Q-score of sharpened map
		nonalignment classification / the final stack			
TRPA1	95.44%	4.29Å/4.34Å	3.96Å/3.89Å	0.41/0.41	0.46/0.46
hemagglutinin	50.66%	4.25Å/4.47Å	3.82Å/4.11Å	0.40/0.35	0.41/0.41
LAT1	67.51%	3.76Å/3.88Å	3.23Å/3.36Å	0.52/0.50	0.53/0.51
pfCRT	96.07%	3.88Å/3.88Å	3.68Å/3.68Å	0.50/0.50	0.51/0.51
TSHR-Gs	97.72%	3.80Å/3.65Å	3.10Å/3.12Å	0.47/0.46	0.47/0.47
TRPM8	94.17%	3.46Å/3.49Å	3.10Å/3.04Å	0.53/0.53	0.57/0.60
apoferritin	59.16%	1.96Å/1.98Å	1.84Å/1.89Å	0.84/0.82	0.89/0.89
streptavidin	99.89%	3.46Å/3.46Å	3.15Å/3.15Å	0.58/0.58	0.61/0.61

Supplementary Table 4: Comparison of the density map of hemagglutinin, LAT1, and apoferritin, derived from non-alignment classification and CryoSieve's finest subsets.

dataset	model to map FSC resolution	half maps FSC resolution	Q-score of raw map	Q-score of sharpened map
	nonalignment classification / the finest subset by CryoSieve			
hemagglutinin	4.25Å/4.19Å	3.82Å/3.62Å	0.40/0.41	0.41/0.44
LAT1	3.76Å/3.69Å	3.23Å/3.11Å	0.52/0.53	0.53/0.53
apoferritin	1.96Å/1.96Å	1.84Å/1.81Å	0.84/0.87	0.89/0.92

Supplementary Table 5: Performance of nonalignment classification on eight experimental datasets.

dataset	parameters of nonalignment classification†	nonalignment classification retaining ratio	model to map FSC resolution	half maps FSC resolution	Q-score of raw map	Q-score of sharpened map
			nonalignment classification / the final stack			
TRPA1	(40, 40, 8)	85.04%	4.34Å/4.34Å	3.96Å/3.89Å	0.41/0.41	0.47/0.46
hemagglutinin	(40, 80, 6)	40.64%	4.36Å/4.47Å	3.79Å/4.11Å	0.39/0.35	0.41/0.41
LAT1	(20, 25, 8)	39.78%	3.80Å/3.88Å	3.20Å/3.36Å	0.52/0.50	0.53/0.51
pfCRT	(20, 40, 6)	98.79%	3.88Å/3.88Å	3.68Å/3.68Å	0.50/0.50	0.51/0.51
TSHR-Gs	(20, 25, 6)	89.53%	3.80Å/3.65Å	3.14Å/3.12Å	0.47/0.46	0.47/0.47
TRPM8	(40, 40, 6)	76.53%	3.46Å/3.49Å	3.03Å/3.04Å	0.53/0.53	0.57/0.60
apoferritin	(20, 25, 8)	63.61%	1.96Å/1.98Å	1.82Å/1.89Å	0.84/0.82	0.89/0.89
streptavidin	(40, 40, 6)	58.91%	3.55Å/3.46Å	3.16Å/3.15Å	0.57/0.58	0.59/0.61

†(a,b,c): (number of classes, number of iterations, tau2_fudge)

Supplementary Table 6: Comparison of the density map of hemagglutinin, LAT1, and apoferritin, derived from non-alignment classification and CryoSieve's finest subsets.

dataset	model to map FSC resolution	half maps FSC resolution	Q-score of raw map	Q-score of sharpened map
	nonalignment classification / the finest subset by CryoSieve			
hemagglutinin	4.36Å/4.19Å	3.79Å/3.62Å	0.39/0.41	0.41/0.44
LAT1	3.80Å/3.69Å	3.20Å/3.11Å	0.52/0.53	0.53/0.53
apoferritin	1.96Å/1.96Å	1.82Å/1.81Å	0.84/0.87	0.89/0.92

Supplementary Material VI: Parameter configurations for CryoSieve and the comparative algorithms.

NCC and random methods do not require specific parameters, relying solely on the retention ratio during each iteration. For cisTEM, it provides a score for every individual particle image. These particle images are then sorted based on their cisTEM score, ensuring the retained particle count is consistent with those in NCC and random for each iteration.

For CryoSieve, NCC, cisTEM, and random, the retention ratio is consistently set to 80% for every iteration.

For AGC and non-alignment methods, the overall retain/removal ratio is intrinsically determined by the algorithms themselves. Both algorithms were evaluated using their default settings.

For CryoSieve, during each iteration, a highpass cutoff frequency needs to be specified. In our implementation, the cutoff frequency changes linearly across iterations, as listed in Supplementary Table 7.

Supplementary Table 7: Parameters of CryoSieve on eight experimental datasets for each iteration

dataset	iter 0	iter 1	iter 2	iter 3	iter 4	iter 5	iter 6	iter 7	iter 8	iter 9	retention ratio
TRPA1	40.0Å	16.9Å	10.7Å	7.8Å	6.2Å	5.1Å	4.3Å	3.8Å	3.3Å	3.0Å	80%
hemagglutinin	40.0Å	16.9Å	10.7Å	7.8Å	6.2Å	5.1Å	4.3Å	3.8Å	3.3Å	3.0Å	80%
LAT1	50.0Å	18.2Å	11.2Å	8.0Å	6.3Å	5.2Å	4.4Å	3.8Å	3.3Å	3.0Å	80%
pfCRT	40.0Å	16.9Å	10.7Å	7.8Å	6.2Å	5.1Å	4.3Å	3.8Å	3.3Å	3.0Å	80%
TSHR-Gs	40.0Å	16.9Å	10.7Å	7.8Å	6.2Å	5.1Å	4.3Å	3.8Å	3.3Å	3.0Å	80%
TRPM8	40.0Å	16.9Å	10.7Å	7.8Å	6.2Å	5.1Å	4.3Å	3.8Å	3.3Å	3.0Å	80%
apoferritin	40.0Å	12.9Å	7.7Å	5.5Å	4.2Å	3.5Å	2.9Å	2.5Å	2.2Å	2.0Å	80%
streptavidin	40.0Å	16.9Å	10.7Å	7.8Å	6.2Å	5.1Å	4.3Å	3.8Å	3.3Å	3.0Å	80%

Supplementary Material VII: Assessing the quality of particles discarded by CryoSieve.

Half-map FSC resolution and Rosenthal-Henderson B-factor were employed to evaluate the quality of the particles discarded by CryoSieve and to determine the amount of information retained in these particles (Supplementary Table 8). Experimental results indicate that these discarded particles have a lower FSC resolution and a higher Rosenthal-Henderson B-factor. We infer that while some information remains in these discarded particles, it primarily serves to interfere with the information present in the finest subset.

Supplementary Table 8: Comparison of Rosenthal Henderson B factor and FSC resolution of futile particles and all particles.

dataset	Rosenthal-Henderson B-factor of futile particles	Rosenthal-Henderson B-factor of all particles	FSC resolution of futile particles	FSC resolution of all particles
TRPA1	348.0Å^2	198.5Å^2	5.97Å	3.89Å
hemagglutinin	878.9Å^2	226.9Å^2	7.01Å	4.11Å
LAT1	469.4Å^2	147.3Å^2	6.05Å	3.36Å
pfCRT	882.0Å^2	235.8Å^2	10.26Å	3.68Å
TSHR-Gs	685.7Å^2	96.9Å^2	8.19Å	3.12Å
TRPM8	169.0Å^2	110.1Å^2	3.77Å	2.96Å
apoferritin	92.9Å^2	81.6Å^2	2.16Å	1.89Å
streptavidin	148.9Å^2	90.4Å^2	3.99Å	3.15Å

Supplementary Material VIII: Performance of various sorting algorithms on simulated radiation damage dataset

We generated simulated particle images using InSilicoTEM [1], with varying degrees of radiation damage, and screened them using CryoSieve.

The atomic model of *E. coli* 50S ribosome bound to VM2 (PDB 6PCQ) [2] was employed to generate particle images, with defocus ranges from 1800 nm to 2800 nm, an electron dose of 40 $e^{-}\mathring{A}^{-2}$, a Falcon I detector, a box size of 450×450, and a pixel size of 1.04Å. Radiation damage was simulated by varying the motion blur factor from 0 to 4 (Supplementary Figure 10a). Each set of the simulated single particles contained 8,000 particles, and the projection directions followed a random distribution.

We evaluated the retention behavior of CryoSieve and NCC for particles with varying simulated degrees of radiation damage, using random retention as a baseline for comparison. With an increase in the number of iterations, the retention rate declined, and CryoSieve displayed a superior ability to find particles with higher levels of radiation damage (Supplementary Figure 10b). While NCC surpassed the random retention baseline in performance, it still fell short of CryoSieve's screening accuracy (Supplementary Figure 10b). Additionally, we compared CryoSieve with cisTEM and found that, although cisTEM performed acceptably for particles with high radiation damage, it was less effective than CryoSieve for particles with relatively low radiation damage (Supplementary Figure 11). CryoSieve achieved higher accuracy than particle selection based on the cisTEM score, especially for particles with relatively low radiation damage (Supplementary Figure 11a). CryoSieve also outperformed cisTEM, as measured by the model-to-map FSC of the reconstructed density map using the retained particles (Supplementary Figure 11b). These results imply that CryoSieve could effectively removes radiation-damaged particles to improve the quality of the final density maps.

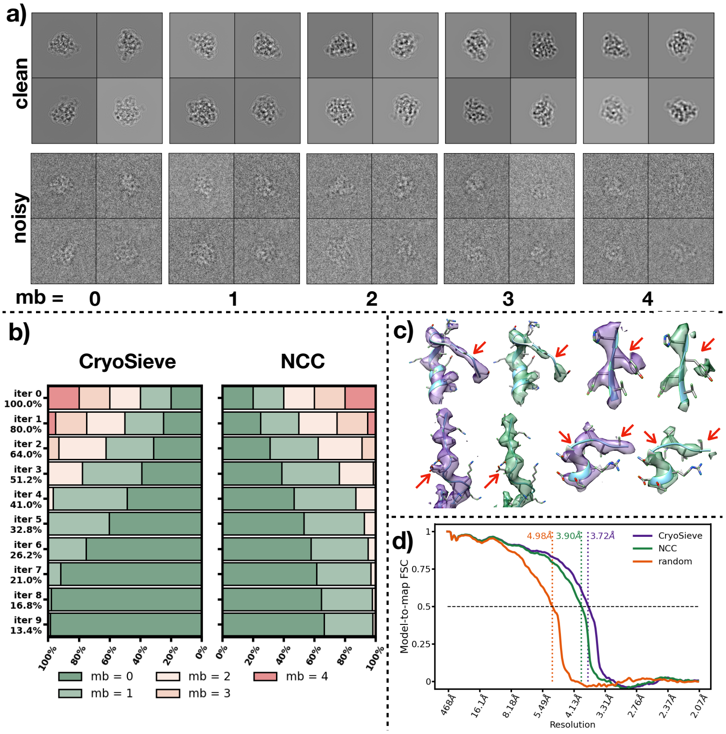

Supplementary Figure 10: **CryoSieve prioritizes the removal of radiation-damaged particles. (a)**, InSilicoTEM generated simulated particle images with different levels of radiation damage. The parameter M_b in InSilicoTEM, ranging from 0 to 4, mimicked different levels of radiation damage, with higher M_b leading to weaker high-frequency signals and blurrier particles (clean, upper row). Noise, also generated by InSilicoTEM through physical process simulation, was then added to the clean particles to obtain noisy particles for testing (noisy, lower row). **(b)**, The proportions of particles with varying levels of radiation damage (distinguished by colors) in retained particles at different retention ratios (labeled on the left) are shown. The retained particles obtained by CryoSieve (left horizontal bars) and NCC (right horizontal bars) were compared. NCC performed acceptably for particles with high radiation damage, but was unable to distinguish particles with relatively low radiation damage. Meanwhile, CryoSieve sequentially sieved out particles from high to low radiation damage. The accuracy (the proportion of zero radiation damage particles) of CryoSieve was 91.7% at iteration 7, while that of NCC was only 61.5%. **(c)**, Side chains of density maps reconstructed by CryoSPARC using retained particles obtained by CryoSieve (indigo) and NCC (green) were compared. The atomic model (PDB 6PCQ) was fitted into the density maps. The two reconstruction maps are compared at an identical contour. Red arrows emphasize differences between the two maps. **(d)** Model-to-map FSCs of reconstructed density maps using retained particles were compared, with retention ratio of 21.0% (iteration 7). Retention particles were obtained by CryoSieve (indigo), NCC (green) and random (baseline method, orange), respectively. The FSC threshold (FSC = 0.5) was depicted as a horizontal dashed line.

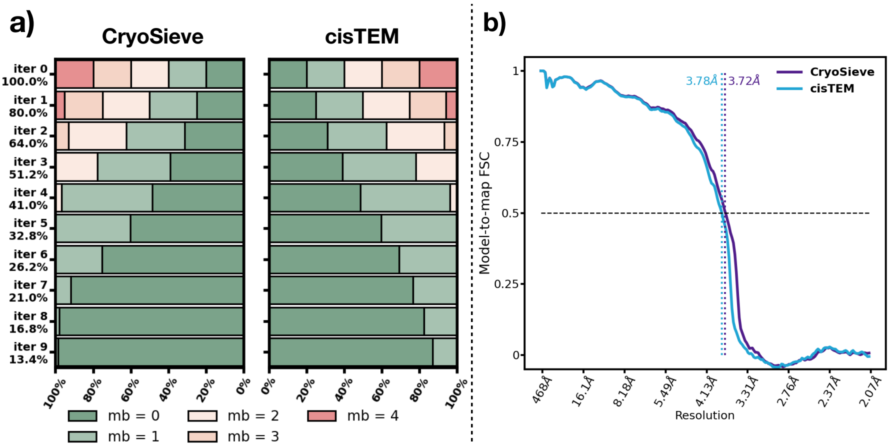

Supplementary Figure 11: **CryoSieve outperformed cisTEM in the removal of radiation-damaged particles. (a)**, At different retention ratios (labeled on the left), the proportions of particles with varying levels of radiation damage (distinguished by colors) in retained particles are shown. The retained particles obtained by CryoSieve (left horizontal bars) and cisTEM (right horizontal bars) were compared. CisTEM performed acceptably for particles with high radiation damage, but still performed inferiorly to CryoSieve for particles with relatively low radiation damage. The accuracy, i.e., the proportion of zero radiation damage particles, of CryoSieve was 91.7% at iteration 7 (rention ratio is 21.0%), while cisTEM's accuracy was only 76.7%. **(b)**, Model-to-map FSCs of the reconstructed density maps using the retained particles were compared. The retention ratio is 21.0% (iteration 7), and retained particles were obtained by CryoSieve (indigo) and cisTEM (blue). The FSC threshold (FSC = 0.5) was depicted as a horizontal dashed line. The FSC of CryoSieve was higher than that of cisTEM, indicating that CryoSieve was more effective in retaining particles with higher resolution information.

References

[1] Vulović, M., Ravelli, R.B.G., van Vliet, L.J., Koster, A.J., Lazić, I., Lücken, U., Rullgård, H., Öktem, O., Rieger, B.: Image formation modeling in cryo-electron microscopy. Journal of Structural Biology **183**(1), 19–32 (2013). https://doi.org/10.1016/j.jsb.2013.05.008. Accessed 2023-02-25

[2] Li, Q., Pellegrino, J., Lee, D.J., Tran, A.A., Chaires, H.A., Wang, R., Park, J.E., Ji, K., Chow, D., Zhang, N., Brilot, A.F., Biel, J.T., van Zundert, G., Borrelli, K., Shinabarger, D., Wolfe, C., Murray, B., Jacobson, M.P., Mühle, E., Chesneau, O., Fraser, J.S., Seiple, I.B.: Synthetic group A streptogramin antibiotics that overcome Vat resistance. Nature **586**(7827), 145–150 (2020). https://doi.org/10.1038/s41586-020-2761-3. Number: 7827 Publisher: Nature Publishing Group. Accessed 2023-02-25
